# DMSU-Net++: A dual multiscale retinal vessel segmentation method based on improved U-Net++

**DOI:** 10.1371/journal.pone.0325625

**Published:** 2025-07-02

**Authors:** Liu Ming, Li Qi

**Affiliations:** School of Electronic and Information Engineering, University of Science and Technology Liaoning, Anshan, Liaoning, China; South China University of Technology, CHINA

## Abstract

Retinal blood vessels are of different sizes and shapes, and even contain very fine capillaries with complex structural morphology, making accurate segmentation a difficult task. To address the above problems, we propose an improved retinal segmentation method DMSU-Net++ (Double Multiscale U-Net++) based on U-Net++. The method innovatively introduces a multiscale feature extraction module WTSAFM, which realises multiscale feature extraction via wavelet transform, and can capture image information of different frequencies more effectively while enhancing global context understanding. In addition, a dual multi-scale feature extraction module is constructed by cascading MFE modules to compensate for the spatial lack of information in WTSAFM, which further improves the accuracy of the model in dealing with different scale information. This method is experimented on the proposed method on two publicly available retinal vessel segmentation datasets, DRIVE and CHASE-DB1, and the experiments show that the F1-score of this method on the two datasets is 82.75 and 82.81, the Sensitivity is 83.74 and 85, and the AUC is 97.86 and 98.36, respectively. Compared with other methods, the method shows better segmentation performance with better accurate recognition.

## Introduction

Retinal Vessels [1] are important in the diagnosis and screening of cardio-ophthalmic diseases such as stroke, venous obstruction, diabetes and atherosclerosis. By analyzing the morphological characteristics of retinal vessels, timely detection and treatment can be achieved in the early stages of disease. The status of retinal vessels in fundus images is an important biomarker for diabetes, hypertension and many ophthalmic diseases. Accurate segmentation of retinal fundus images can assist in the diagnosis of diabetic retinopathy, glaucoma, and age-related macular degeneration. First, retinal blood vessels vary in size and thickness, shape, and complexity. Second, the contrast between the vessels and the background is low, especially at vessel crossings and vessel endings. Further, when color retinal vessel images are acquired, the illumination is uneven and the available dataset is small. Therefore, it is challenging to segment retinal vessels quickly and efficiently.

With the continuous progress of deep learning technology, domestic and foreign scholars have proposed many methods for retinal vessel segmentation. LadderNet [2] is a multipath network architecture based on U-Net, which realizes multipath information flow by stacking multiple U-Net structures into a “ladder” shape and combining them with residual connectivity to enhance feature extraction and reuse. CE-Net [3] enhances the feature extraction capability of the model by introducing residual block, ASPP module, and Context Encoder. LKR-Unet uses large kernel residual convolution block (IKR-Block), which is the most powerful and efficient way to extract features. Block (IKR-Block) and cascaded spatial channelattention (CSCA) to improve the accuracy of segmentation. Zhou et al. [4] proposed U-Net++, which introduces a nested U-Net structure and dense hopping connections to enable the network to aggregate different levels of feature information more efficiently. U-Net++ is useful in processing features with different levels of complexity. -Net++ is able to capture small objects, fuzzy boundaries and complex structures in images more accurately when processing medical images with complex structures and subtle features.

Although U-Net, U-Net++ and other U-shaped networks have achieved remarkable results in the field of blood vessel segmentation, there are still problems. Retinal blood vessels are complex and diverse, and a single scale will inevitably lead to the loss of detailed information in blood vessel images; the detection of fine blood vessels is easily interfered by background noise, leading to false or missed detection. To address these problems, a new network structure model DMSU-Net++ (double multi-scale U-Net++) is proposed for retinal blood vessel image segmentation based on UNet++.

## Related work

### U-Net++

U-Net++ is an improved convolutional neural network (CNN) based on the U-Net architecture, mainly for biomedical image segmentation tasks. The main innovation of U-Net++ compared to the original U-Net is the introduction of nested jump connections. These jump connections are connected through multiple intermediate nodes, allowing the model to capture a more diverse set of features.

U-Net++‘s nested jump-join structure allows the output of each submodule to be passed not only to the next layer, but also to the subsequent decoder layer, thus increasing the efficiency of feature reuse. This design allows U-Net++ to produce finer segmentation results. Nested jump connections also enhance the robustness of the model to targets of different sizes, as they allow the model to reuse and integrate features at different layers, thus improving the accuracy and detailed performance of the segmentation.

### Feature grouping

The importance of feature grouping in deep learning has been widely researched and verified. AlexNet [5] introduces grouped convolution for the first time, which divides the convolution operation into two groups, reduces the computational burden of a single GPU, and realizes multi-GPU parallel processing. It improves the computational efficiency and reduces the number of model parameters, which helps to reduce the risk of overfitting. In ShuffleNet [6], grouped convolution is also used to reduce the number of parameters and computation of the model, and through an efficient channel mixing strategy, ShuffleNet reduces the complexity of the model while maintaining the performance. ResNeXt [7] further develops on the basis of feature grouping by increasing the number of feature groups. This activation of multiple groups of features better adapts to different image patterns and background noise, thus improving the robustness and generalization ability of the model. EMA [8] simulates the correlation between local and global attentional information by dividing the channel dimension into multiple groups and introducing two parallel branches. By grouping channels, each sub-feature group can learn different aspects of the data or features at different scales, which helps the model to capture richer information and enhance feature representation.

### Multiscale convolution

The application of multi-scale convolution in convolutional neural networks (CNNs) greatly enriches the feature representation capability and improves the performance of the model in processing complex image tasks. Inception [9] network is able to capture multi-scale spatial information in the same processing stage by designing a multi-branch structure with different sized convolution kernels in each branch. This design allows the network to perform feature extraction on different receptive fields, enabling the model to fuse features from different scales, greatly enhancing the diversity and richness of the feature space. The selection kernel network SKNet [10] introduces an adaptive selection strategy that allows neurons to dynamically adjust the size of their receptive fields. Through this adaptive mechanism, the network can automatically select the optimal convolution kernel size according to the features of the input image, thus processing information of different scales more flexibly and enriching the feature representation effectively. A multi-scale contextual feature fusion network based on cavity convolution is proposed in MCANet [11], which solves the problem of the change of the size and scale of the object by fusing the multilayer features of the cavity convolution and adding the detection header that significantly improves inference speed and average accuracy. EPSANet [12] employs a multi-scale pyramid structure instead of the traditional 3x3 convolution to model cross-channel interactions in a localized manner. This design allows the model to independently learn spatial information at different scales, further enhancing the feature representation. With the multi-scale pyramid structure, the features extracted by EPSANet at different scales can complement each other, enhancing the overall performance of the model.

### Feel the wild

There are many practices to expand the perceptual field in CNN, such as VGG [13] network using network depth to expand the perceptual field, increasing the depth of the model can expand the perceptual field to a certain extent, but it will greatly increase the number of model parameters, and the effect of expanding the effective perceptual field is not very good; the large kernel convolution can significantly increase the perceptual field of the convolutional layer, and the large kernel of single convolutional neuron has a wider perceptual range, covering a larger image region. The ability of large kernel convolution to increase the perceptual field with fewer parameters may also pose some challenges such as increased computational cost and risk of overfitting. Hollow Convolution [14] of input feature maps in larger steps, thus expanding the perceptual field without increasing the number of parameters. FPN [15] Combining high-resolution shallow features with low-resolution deep features through top-down paths and lateral connections can capture multi-scale features, thus expanding the perceptual field. Wavelet transform is widely used in the field of signal processing, which has been applied in neural networks in recent years due to its ability to effectively decompose the different frequency components of the input and operate at multiple frequency levels to maintain localized information, thus enhancing the performance of neural networks. For example, Duan et al. [16] and Williams and Li [17] used wavelet transform to perform pooling operation in the network. Liu et al. [18] introduced wavelet transform in U-Net architecture for downsampling and upsampling. Wavelet Compression Convolution (WCC) [19] Combining Haar wavelet transform with convolution effectively reduces the computational resource requirements for convolution in image-to-image tasks.

## DSMU-Net++

### General network architecture

Despite its remarkable achievements in image segmentation, there is still room for further optimization. U-Net++ enhances the information richness of the decoder by adding jump connections, but these connections may not fully preserve the multi-scale contextual information in the encoder stage. In image segmentation, multi-scale information is crucial for accurately recognizing and localizing objects of different sizes and shapes.

To overcome this challenge, multi-scale feature fusion modules can be introduced at the encoder stage. These modules enhance the ability of the encoder to capture multi-scale information by merging feature maps from different scales.

This paper proposes DMSU-Net++ based on UNet++. By designing the cascaded multi-scale module DMS, the aggregation of features is performed many times to extend the perceptual range of the network and strengthen the feature extraction capability. With this strategy, U-Net++ not only retains more contextual information, but also handles objects of various scales more flexibly, further improving its performance in image segmentation tasks. This improvement helps the model to recognize and segment targets more accurately when facing complex scenes.

**Fig 1:**
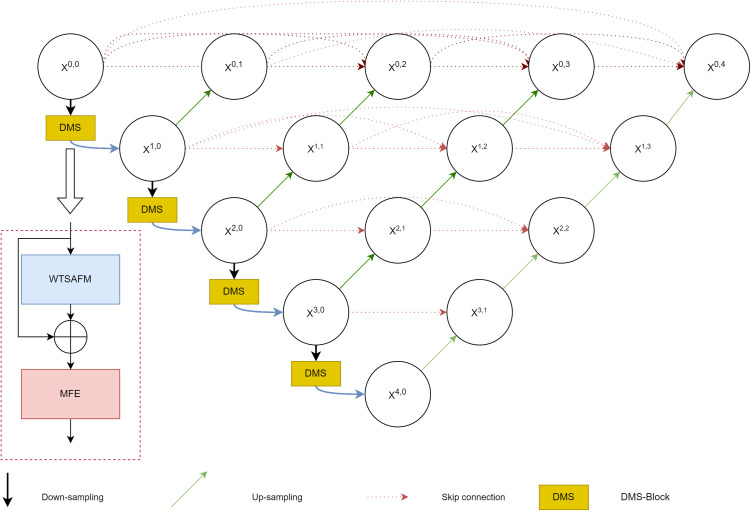
DMSU-Net++ network general architecture.

The basic network architecture of the DMSU-Net++ model is shown in Fig 1, which adopts the improvement of the encoder part on the basis of UNet++, and firstly, the image features are feature extracted by a wavelet spatial adaptive adjustment module (WTSAFM), which performs different degrees of downsampling, and extracts different information of the image on different scales, and then they are combined by the multiscale module (MFE) to combine them together. This design helps the U-Net++ architecture to coordinate the features learned from images at different scales, which further enhances the generalization ability of the network and the representation of the network structure.

### Wavelet spatial adaptive fitting module

UNet++ employs fixed convolution and pooling operations to generate features for multi-scale feature extraction, this approach makes the network only capture predefined scale information and cannot adaptively adjust the scale of feature extraction. Therefore, it will be insufficient in dealing with images with more complex or dynamically changing scales. In addition, the convolutional operation of UNet++ focuses mainly on localized regions and the sensory field is fixed, resulting in a network that lacks the ability to capture the global context of an image. In this way, when dealing with images with extensive contextual dependencies or where the overall structure is important, the segmentation effectiveness of UNet++ may be limited because it is unable to effectively integrate global information to aid in the understanding of local features.

**Fig 2:**
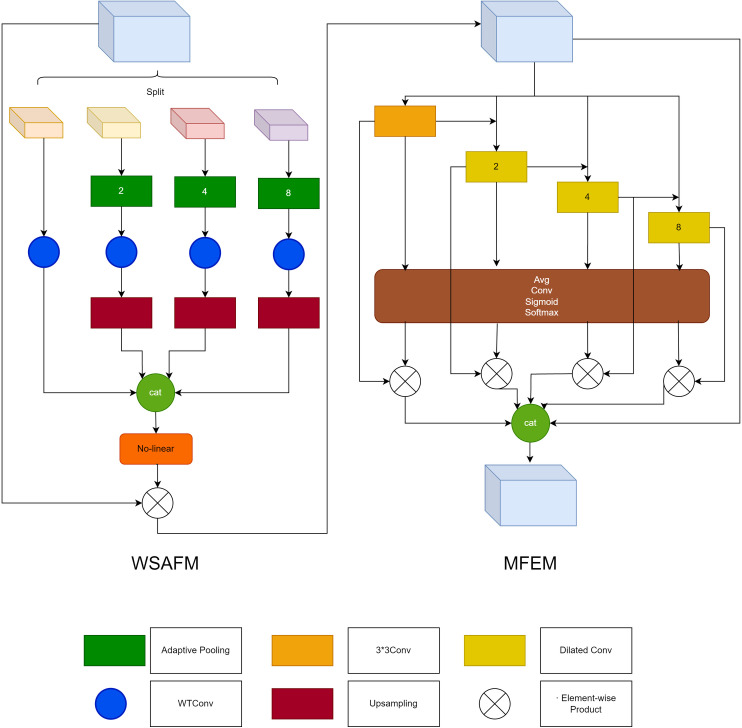
DMS module structure.

As shown in Fig 2 left, the main structure of the WTSAFM module is formed by combining the SAFM [20] module with wavelet convolution, the main purpose of which is to use feature grouping to reduce the complexity of the model and to use the maximum pooling layer with different window sizes to generate multi-scale features, and then to integrate wavelet convolution on the base of the module to further improve the feature extraction capability.

The normalized input features are first subjected to a channel segmentation operation to produce four parts of molecular features. This four-part feature is processed differently, only 3 × 3 wavelet convolution is performed for the first part to retain the global information; for the rest of the parts, pooling operations are performed using convolution kernels of different scales to extract the local features to different scales, which are then recovered to the original size by up-sampling after a layer of 3 × 3 wavelet convolution, and then the aggregated multiscale features are aggregated. After obtaining the aggregated features, they are normalized by GELU activation function to estimate the attention. And adaptive modulation of X is performed based on the estimated attention as a product of elements. Residual connectivity is also introduced, but unlike before it is realized by element-by-element multiplication. While preserving the original features, multi-scale local features are fused to enrich the output features.

### Multiscale feature extraction module

Since the Wavelet Spatial Adaptive Fitting Module (WTSAFM) uses maximum pooling layers with different window sizes to generate multiscale features, this operation results in spatial loss of information, so it is proposed to use the Multiscale Feature Extraction (MFE) module to feature extract the image again to mitigate the loss of spatial information. The MFE [21] module is shown in Fig 2 right.

Multi-scale features of the input feature map are first extracted using convolutional layers with different expansions, and links are constructed between the convolutional layers with different expansions so that the features of each layer except the first one contain not only the original input, but also the features of the previous layer. This operation gives each layer access to both the original global information and the local information extracted from the previous layer, thus enhancing the contextual dependency of the features in each layer. Global average pooling of processed multi-scale features is performed to construct channel correlations, and then the global information from multiple scales is spliced together before obtaining weight representations using the Sigmoid activation function. Finally, softmax is used to obtain the weights of each scale, multiply the corresponding normalized weights of each feature, and then sum the processed features to generate new multi-scale features.

### Wavelet convolution

Wavelet Convolution [22] The input image is first decomposed into different frequency components by wavelet transform (WT), and then a small kernel deep convolution operation is applied to the decomposed frequency components to process the low frequency information and high frequency information separately. This operation not only separates the frequency components, but also allows the smaller kernel to operate over a wider range of the original input, i.e., increasing its receptive field to the input. Finally the decomposed frequency components are reconstructed back to the original image by the Inverse Wavelet Transform (IWT). The computational flow can be represented as:


Y=IWT(Conv(W,WT(X))) (1)

The wavelet convolution layer is constructed to capture low frequencies better than standard convolution. This is because repeated WT decomposition of the input low frequencies highlights these low frequencies and increases the corresponding response of the layer.

In summary, wavelet convolution is a combination of wavelet transform and convolution operation, which is capable of achieving a larger sensory field while maintaining efficient feature capture.

## Experiment

### Dataset and data preprocessing

**Fig 3:**
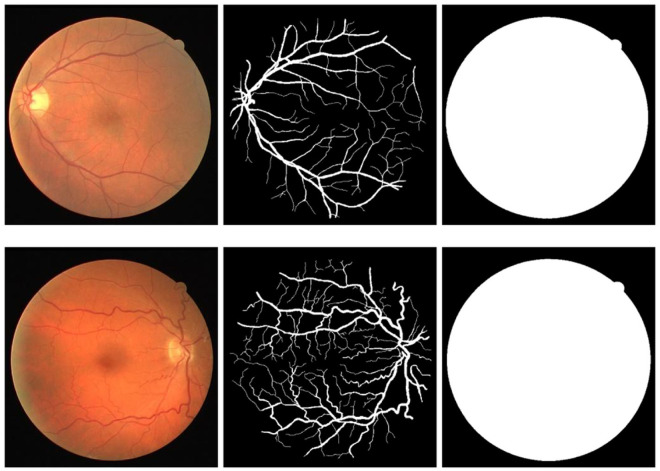
Example image of a portion of the DRIVE dataset.

In this paper, the DRIVE and CHASE-DB1 datasets were selected as the experimental datasets. Both of these datasets are publicly available retinal vessel segmentation datasets. The DRlVE dataset is collected by the Diabetic Retinopathy Screening Program in the Netherlands, which has a total of 40 color fundus images with an image resolution of 584 × 565 pixels, as shown in Fig 3. Each image has segmentation results and masks drawn manually by ophthalmologists, and the dataset is divided into a training set and a test set, each containing 20 images.

**Fig 4:**
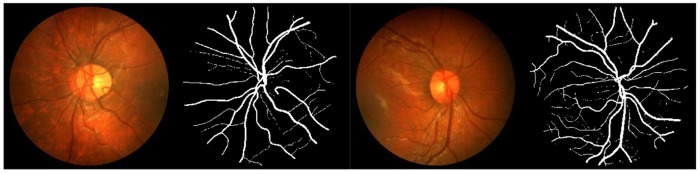
Example image of a portion of the CHASE-DB1 dataset.

The CHASE-DB1 dataset has a total of 28 images from the retinas of the left and right eyes of 14 children, each with a resolution of 999 × 960 pixels. In this paper, the first 19 images are used as the training set and the last 9 images are used as the test set. Some images of the dataset are shown in Fig 4.

**Fig 5:**
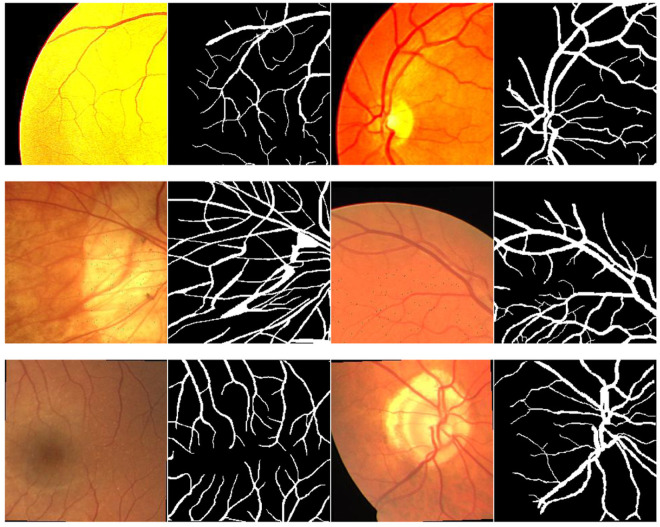
Enhanced image example map.

Due to the small size of the DRIVE and CHASE-DB1 datasets, they cannot meet the demand of data for deep learning. In this paper, four methods are adopted to expand the dataset, including randomly rotating any angle; adding Gaussian random noise; adjusting color saturation, brightness, contrast and sharpness; and randomly cropping by setting the size and step of the cropping region. Taking DRIVE as an example, the expanded image and mask are shown in Fig 5.

### Experimental setup

In this paper, Pytorch was chosen as the core framework of the experimental platform, and the training and testing process was completed on an RTX3090 graphics card equipped with 24GB of video memory. U-Net++ was used as the backbone network for this experiment, and this study made further improvements and optimizations on the basis of this network. In the training phase, the Adam optimizer was chosen to guide the learning process, the batch size was set to 2, the learning rate was initialized to 1e-4, and the training iteration period was set to 100 Epochs.

### Evaluation indicators

The main thrust of the experiments in this chapter is the validation of the image segmentation problem, which belongs to the dichotomy of classification problems. The model outputs a confusion matrix that is used to assign each pixel its probability of belonging to that category. TP (True Positive) is the number of True Positives, the number that are true in the label and also true in the predicted value. TN (True Negative) is the number of True Negatives, the number that are false in the label and also false in the predicted value. FP (False Positive) is the number of False Positives, which are false in the label and true in the predicted value. FN (False Negative) is the number of False Negatives, which are true in the label and false in the predicted value. FN (False Negative) is the number of False Negatives, which are true in the label and false in the predicted value. The experiments in this chapter use four evaluation metrics, F1-score, Specificity and Recall, and Area Under the Roc Curve (AUC), to assess the segmentation quality of the method proposed in this paper on the dataset. The calculation of each metric is shown in Table 1.

**Table 1:** Evaluation indicator.

Assessment of indicators	Formula
F1-score	$\frac{2TP}{2TP+FP+FN}$
Sensitivity	$\frac{TP}{TP+FN}$
Specificity	$\begin{array}{c} \frac{TN}{TN+FP} \end{array}$

### Analysis of results

#### Ablation experiments.

Method1, Method2, and Method3 represent WTSAFM module, MSE module, and DMS module respectively.

The ablation experiments in this paper use U-Net++ as the baseline model, and Method1, Method2, and Method3 are sequentially incorporated into the baseline model for ablation experiments. As shown in Table 2, the U-Net++ model performs poorly in the fine-grained segmentation task, with limited ability to handle target boundaries and details. The Method1 model outperforms U-Net++ in all metrics, especially in SE and SP. Method2 performs on par with Method1 in most of the metrics, with SP slightly lower than that of Method1. Method3 performs well in all the performance indexes and is the optimal choice among the methods in this paper. It is ahead of the other methods in classification accuracy, detection ability and detail processing of the target region, which indicates that Method3 is able to better acquire the feature information of retinal blood vessels.

**Table 2:** Ablation experiments on the DRIVE dataset.

Model architecture	F1-score	Sensitivity (SE)	Specificity (SP)	AUC
U-Net++	80.34	80.61	97.42	97.26
Method1	81.54	83.21	97.75	97.61
Method2	81.75	83.17	97.25	97.68
Method3	82.75	83.74	98.45	97.86

As shown in Table 3, the results of the ablation experiments on the CHASE-DB1 dataset indicate that Method3 performs the best in the retinal vessel segmentation task, especially achieving the highest values for F1-score, SE, and SP, suggesting that it has the best results in accurately identifying and segmenting blood vessels. The performances of Method1 and Method2 are more similar.

**Table 3:** Ablation experiments on the CHASE-DB1 dataset.

Model architecture	F1-score	Sensitivity	Specificity	AUC
U-Net++	80.15	82.26	97.79	97.54
Method1	82.13	83.55	97.78	97.97
Method2	81.90	84.26	97.90	97.28
Method3	82.81	85.0	98.80	98.36

The results of the ablation experiments showed that Method3 improved in all listed performance metrics F1-score, Sensitivity, Specificity, and AUC.

According to the results of the ablation experiments, Method1 improves the ability to capture multi-scale features by introducing feature selection and adaptive mechanisms based on U-Net++. Compared with U-Net++, the F1-score of Method1 is improved by 1.20% and 1.98%, indicating that it can better extract features at different scales, and especially performs better in the recognition of complex blood vessel structures. Method2 strengthens the feature extraction ability through multi-scale feature enhancement, especially the recognition of small blood vessels and detailed parts is enhanced. On the DRIVE dataset, Method2 showed a slight improvement in F1-score and AUC compared to Method1, with an increase of 0.21% and 0.02%, respectively; on the CHASE-DB1 dataset, Method2 showed a decrease of 0.23% in F1-score and an increase of 0.71% in Sensitivity compared to Method1. Method3 combines the above two methods, and applies multi-scale feature enhancement on the basis of the ability of adaptive multi-scale feature selection to have a stronger feature extraction capability, and also better balance the weights between different scale features, which significantly improves the segmentation accuracy and generalization ability, and on the DRIVE dataset compared to the baseline model, F1-score, Sensitivity, Specificity increased by 0.23% and Sensitivity increased by 0.71%, respectively. and Specificity on the DRIVE dataset increased by 2.41%, 3.13%, and 1.03%, respectively; F1-score, Sensitivity, and Specificity on the CHASE-DB1 dataset increased by 2.66%, 2.74%, and 1.01%, respectively.

#### Comparison experiments.

In order to verify the effectiveness and feasibility of the method proposed in this paper on retinal blood vessel segmentation, comparative experiments were conducted on DRIVE and CHASE-DB1 datasets, and the segmentation effects of different networks are shown in Tables 4 and 5.

**Table 4:** Comparison of different methods on the DRIVE dataset.

Methodologies	F1-score	Sensitivity	Specificity	AUC
CE-Net [3]	–	83.09	–	97.79
LMSA-UNet [23]	–	83.08	98.21	98.46
TCCNNet [24]	82.22	82.56	98.25	98.57
LadderNet [2]	82.02	78.56	98.10	97.93
SA-UNet [25]	82.63	82.12	98.40	98.64
DMSU-Net++	82.75	83.74	98.45	97.86

**Table 5:** Comparison of different methods on the CHASE-DB1 dataset.

Methodologies	F1-score	Sensitivity	Specificity	AUC
LMSA-UNet	–	84.28	98.40	98.90
TCCNNet	82.52	78.68	99.12	99.00
LadderNet	80.31	79.78	98.18	98.39
SA-UNet	81.53	85.73	98.35	99.05
DMSU-Net++	82.81	85.0	98.80	98.36

Based on the results presented in the comparison experiments, it can be seen that the method proposed in this paper shows high performance in most of the metrics. This indicates that DMSU-Net++ has the best overall performance in retinal blood vessel segmentation task, especially in F1-score, Sensitivity metrics. The overall analysis shows that the segmentation method in this paper performs well in the retinal blood vessel segmentation task and significantly improves the performance.

**Fig 6:**
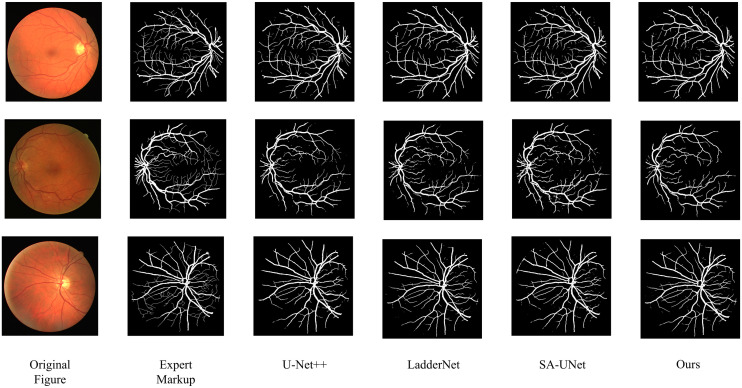
Segmentation results on the DRIVE dataset.

**Fig 7:**
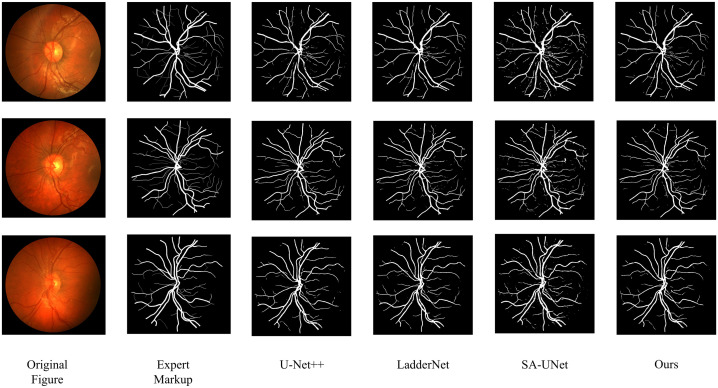
Segmentation results on the CHASEDB1 dataset.

In order to prove the practical effect of this paper’s method, the predicted segmentation is performed on DRIVE and CHASEDB1 datasets respectively, and the actual segmentation results are shown in Figs 6 and 7. On the whole, the proposed method in this paper is better in capturing the fine blood vessel branches of the retina, which is closer to the results labeled by experts, especially in the detail processing of the vessel ends.

## Conclusion

Aiming at the problems of existing algorithms, such as limited feeling field, difficulty in capturing multi-scale vascular features, and insufficient feature fusion and dynamic adjustment ability. Firstly, the introduction of the WTSAFM multiscale feature extraction module can effectively capture various frequency information, thus improving the global background understanding of the model. In addition, the cascaded MFE module in the dual multiscale feature extraction framework compensates for the lack of spatial information and further improves the segmentation accuracy. Relevant experiments on DRIVE and CHASE-DB1 datasets show that the segmentation performance of DMSU-Net++ performs well compared with other networks and is capable of accomplishing the task of accurate segmentation of retinal blood vessels.
